# A versatile isothermal amplification assay for the detection of leptospires from various sample types

**DOI:** 10.7717/peerj.12850

**Published:** 2022-03-10

**Authors:** Shuhaidah Othman, Pui-Yuei Lee, Jia-Yong Lam, Noraini Philip, Nurul Natasya Azhari, Norliza Bahtiar Affendy, Siti Norbaya Masri, Vasantha Kumari Neela, Farah Shafawati Mohd-Taib, Hui-Yee Chee

**Affiliations:** 1 Department of Medical Microbiology, Faculty of Medicine and Health Sciences, Universiti Putra Malaysia, Serdang, Selangor, Malaysia; 2 Department of Biological Sciences and Biotechnology, Faculty of Science and Technology, Universiti Kebangsaan Malaysia, Bangi, Selangor, Malaysia

**Keywords:** Leptospirosis, Loop-mediated isothermal amplification, Clinical detection, Vector surveillance

## Abstract

**Background:**

Leptospirosis is a zoonotic disease caused by bacteria of the genus *Leptospira* that affects both humans and animals worldwide. Early detection of the pathogen in humans is crucial for early intervention and control of the progression of the disease to a severe state. It is also vitally important to be able to detect the presence of the pathogen in carrier animals to control the spread of the disease from the environment. Here we developed a simple and rapid loop-mediated isothermal amplification (LAMP) assay targeting the leptospiral *secY* gene.

**Results:**

Several reaction conditions of the LAMP reaction were optimized to ensure efficient amplification of the target DNA. The sensitivity of the developed LAMP assay obtained using a pure *Leptospira* culture was 2 × 10^4^ copies of genomic DNA per reaction (equivalent to 0.1 ng) for a 40-minute reaction time. No cross-reactions were observed in the LAMP reaction against a series of non-leptospiral bacteria, indicating a specific reaction. The applicability of the LAMP assay was demonstrated on human blood and urine specimens collected from suspected leptospirosis patients and rat kidney specimens collected from suspected leptospirosis outbreak areas and high-risk areas. The developed LAMP assay demonstrated a higher detection rate for leptospiral DNA compared with the polymerase chain reaction (PCR) assay, possibly due to the presence of inhibitory substances, especially in rat kidney specimens, to which the PCR method is more susceptible. The present findings also highlight the importance of urine sample collection from patients for routine monitoring of the disease.

**Conclusions:**

In short, the developed LAMP assay can serve as a feasible alternative tool for the diagnosis of leptospirosis and be used for epidemiological and environmental surveillance of the disease, considering its robustness, rapidity, sensitivity, and specificity, as demonstrated in this study.

## Introduction

Leptospirosis is a disease caused by bacteria of the genus *Leptospira* that affects both humans and animals worldwide. Infection in humans occurs through contact with water, food, or soil contaminated with the urine of infected animals (Levett, 2001). Unlike other reservoir animals such as dogs and cattle, which act as temporary carriers of *Leptospira* for several months, rats usually remain permanent carriers of *Leptospira* throughout their lives (Zenebe, Abdi & Keskes, 2013). The majority of human infections have been attributed to rats and other rodent species, which are known to be a permanent carrier of *Leptospira* spp. Previous outbreaks of leptospirosis have been linked with intense periods of high rainfall, which facilitates the spreading of leptospires shedding in the urban environment (Lee et al., 2021). In humans, a wide range of non-specific symptoms such as high fever, headache, muscle ache, and vomiting may appear, and these are often mistaken for other diseases. In some infected persons, symptoms may occur at the later stage, leading to a much later detection of the disease that could potentially lead to death due to multiorgan system complications (Adler, 2015). Due to this problem, early detection of leptospirosis is crucial in order to provide appropriate treatment and control disease progression to the severe state. Furthermore, it is important that this fatal disease be detected at an early stage because antibiotic therapy is most effective when initiated early (McBride et al., 2005).

To date, there are a few alternatives for leptospirosis diagnosis. Serology is the most widely used diagnostic tool in the detection of this disease. The microscopic agglutination test (MAT) is known as the gold standard for the diagnosis of leptospirosis (Aslan, 2016). However, this approach does not contribute to early diagnosis because anti-*Leptospira* antibodies only become detectable in the late acute phase, 3–5 days after disease onset (Ahmed, Linden & Hartskeerl, 2014). The low sensitivity of the MAT has also been associated with a low number of antibodies in the early stage of infection (Niloofa et al., 2015). Dark field microscopy is used to view the organism in urine or blood; this is an example of a low-cost diagnostic tool. However, this method requires the specimen to be prepared from a culture, which is impractical due to the fastidious nature of the bacteria (Adler & de la Peña Moctezuma, 2010), in addition to the low reported specificity (Ahmad, Shah & Ahmad, 2005; Rodríguez et al., 2013). Genomic methods, including polymerase chain reaction (PCR), multiplex PCR, and real time PCR, are among the most reliable tools in terms of sensitivity and rapidity of detection. Nevertheless, the need for an expensive thermocycler, which may not be readily available in many laboratories of resource-limited countries, has become a major drawback of this diagnostic method. Currently, no diagnostic technique is completely satisfactory, and the absence of an adequate laboratory test remains a key barrier to the diagnosis of this disease (Toyokawa, Ohnishi & Koizumi, 2011).

Cost effective alternatives for rapid detection with a high degree of reliability and sensitivity may become the ultimate solution for the control of this infectious disease (Saharan et al., 2014). Notomi et al. (2000) reported a novel molecular technique of nucleic acid amplification, termed loop-mediated isothermal amplification (LAMP), where a set of four (or six) different primers bind to six (or eight) different regions on the target gene making it highly specific (Notomi et al., 2000). In addition, the use of *Bst* DNA polymerase with high-displacement activity enables the LAMP reaction to be performed at a constant temperature, simply by using a water bath (Notomi et al., 2000). In terms of cost effectiveness, this method does not require an expensive instrument, such as a thermocycler, which is needed in conventional PCR (Koizumi et al., 2012). Various LAMP detection methods, such as turbidimeters, fluorescence agents, colorimetric agents, lateral flow dipsticks, and lab-on-a-chip devices, have been developed (Nurul Najian et al., 2016).

For the past decade, a growing number of studies have been conducted to assess the application of the LAMP method to the detection of *Leptospira* spp. in a variety of biological samples (clinical blood samples, urine specimens, simulated blood and urine samples, and genomic DNA isolated from culture) targeting different genes (*rrs*, *lipL32*, *lipL41*, and *ligB*) (Chen et al., 2015; Koizumi et al., 2012; Lin et al., 2009; Sengupta et al., 2017; Sonthayanon et al., 2011; Suwancharoen, Sittiwicheanwong & Wiratsudakul, 2016). As reported by Suwancharoen, Sittiwicheanwong & Wiratsudakul (2016), the LAMP method provides a specific assay 10–100 times more sensitive than standard PCR in the detection of *Leptospira* spp. (Suwancharoen, Sittiwicheanwong & Wiratsudakul, 2016). The leptospiral *secY* gene located in the S10-spc- *α* locus containing genes for ribosomal proteins, encodes for the leptospiral preprotein translocase, a processive enzyme (Zuerner et al., 2000). As such, the *secY* gene is a housekeeping gene that it is more likely to be present in all *Leptospira* species and strains regardless of their pathogenicity group (Ahmed et al., 2009; Salgado et al., 2015). The *rrs* gene encodes for the highly conserved 16S rRNA ribosomal subunit that is also ubiquitously present in all *Leptospira*. However, most of the previous studies have focused on the LAMP detection of the pathogenic and intermediate groups of pathogens by targeting the *lipL32*, *lipL41* or *ligB* gene (Koizumi et al., 2012; Lin et al., 2009; Suwancharoen, Sittiwicheanwong & Wiratsudakul, 2016). Both *lipL32* and *lipL41* encode for two major lipoproteins located in the outer membrane of *Leptospira* (King et al., 2013; Thibeaux et al., 2018) while *ligB* encodes one of the proteins in the leptospiral immunoglobulin-like protein family (Matsunaga et al., 2003). These genes serve unique virulence factors that are only found in pathogenic leptospires but absent in saprophytic species (Fouts et al., 2016). However, it was reported that saprophytic strains of *Leptospira* had also been detected in patients (Bedir et al., 2010; Richtzenhain et al., 2002; Xiao et al., 2015). The development of a leptospirosis diagnostic test that focuses on the detection of pathogenic strains may cause a nonresponsive result or false-negative outcome (Alizadeh et al., 2016). Therefore, it would only seem plausible to design an assay that can target all three groups of *Leptospira*. Hence, in this study we developed a sensitive and robust LAMP assay targeting the *secY* gene of *Leptospira* and performed an analytical validation of its applicability on human specimens collected from suspected leptospirosis patients and rat kidney specimens collected from suspected leptospirosis outbreak areas and high-risk areas.

## Methods

### Clinical patient specimens

Blood and urine samples were collected from suspected leptospirosis patients in Hospital Serdang, Selangor, Malaysia and Hospital Tengku Ampuan Rahimah, Selangor, Malaysia from June 2016 until December 2017 during admission to the ward and upon discharge from the hospitals. Written informed consent was obtained from all subjects participating in this study. Ethical approval was obtained from the Medical Research and Ethics Committee, Ministry of Health Malaysia (Reference number: NMRR-15-2148-27536).

### Kidney specimens from rats

Rat kidney samples used in the present study were obtained from previous studies (Azhari et al., 2018; Bahtiar Affendy et al., 2020) in which ethical approval has been obtained from the Animal Ethics Committee of the Universiti Kebangsaan Malaysia (Reference number: (UKMAEC) FST/2016/AR-CAT2). The rat kidney samples utilized in the present study originated from two types of areas defined in the previous studies, suspected leptospirosis outbreak area and high-risk areas. Samples from suspected leptospirosis outbreak area were captured from selected sites of urban, semi-urban and forest site in Selangor, Malaysia (Azhari et al., 2018). Meanwhile, samples from high-risk areas were captured from wet markets located in Seri Kembangan, Selangor, Malaysia and Bangi, Selangor, Malaysia (Bahtiar Affendy et al., 2020). The animal study method has been described extensively by Azhari et al. (2018).

### Primer design

The gene sequences of the *Leptospira secY* gene (EU357956.1, EU357961.1, EU357997.1, EU358012.1, EU358013.1) were retrieved from NCBI nucleotide database (https://www.ncbi.nlm.nih.gov/nucleotide/). Multiple sequence alignment was performed using Clustal Omega (https://www.ebi.ac.uk/Tools/msa/clustalo/). To design the LAMP primers, OptiGene LAMP Designer software (http://www.optigene.co.uk/lamp-designer/) was used. The LAMP primer set consists of two outermost primers (F3 and B3), two inner primers (FIP and BIP), and two loop primers (LF and LB) (Lee et al., 2021). The primer sequences are listed in Table 1 and their respective mapped regions are shown in Appendix B. All of the primers used in this study were synthesized by Integrated DNA Technologies, Singapore.

### DNA extraction

Genomic DNA of pure *Leptospira* cultures and other bacterial DNA was extracted using the Wizard^®^ Genomic DNA Purification Kit (Promega, USA). For human samples, genomic DNA from blood samples was extracted using the QIAamp DNA Blood Mini Kit (QIAGEN, Germany) while the GeneMATRIX Bio-Trace DNA Purification Kit (EURx, Poland) was used to extract genomic DNA from urine samples. The direct lysis method was also utilized to extract the DNA from spiked urine samples for comparison against the commercial kit. The protocol for direct lysis was adapted from Koizumi et al. (2012), with modifications (Koizumi et al., 2012). Briefly, one ml of spiked urine was centrifuged at 16, 000 × g for 10 min, and the resulting pellet was resuspended in 20 µl sterile water. The suspension was then boiled at 100 °C for 10 min, followed by centrifugation at 16, 000 × g for 10 min, to which the supernatant was retained for use in LAMP. Genomic DNA of rat kidneys was extracted using the FavorPrep™ Tissue Genomic DNA Extraction Mini Kit (Favorgen Biotech, Taiwan). All the extraction procedures were performed in accordance with the manufacturer’s procedures. The concentration and purity of the extracted DNAs were quantified using a NanoDrop™ ND-1000 spectrophotometer (Thermo Fisher Scientific, USA). The samples were kept at −20 °C until further use.

**Table 1:** List of primers used in this study.

Primer	Sequence (5′–3′)
LAMP-secY-F3	CTTGTTCCTGCCCTTCAAA
LAMP-secY-B3	TTCGGTGATCTGTTCTCCT
LAMP-secY-FIP	TTCCGTGCCGGTAGACCAGAACCGTAATTCTTTGTGCG
LAMP-secY-BIP	CTTGAGCCTGCGCGTTACAATGAGAAGAACGGTTCCG
LAMP-secY-LF	GCGAGTTGGATCACTGCTA
LAMP-secY-LB	CCGGGCTTAATCAATTCTTCTG

### Optimization of *secY* LAMP assay conditions

The LAMP reaction was initially performed using the Loopamp DNA Amplification Kit (Eiken Chemical, Japan) to optimize the reaction temperature. The self-assembled 2 × LAMP reaction mixture consists of *2x* ThermoPol^®^ buffer (New England Biolabs, Ipswich, MA, USA), 12 mM of magnesium sulfate, MgSO_4_ (New England Biolabs, Ipswich, MA, USA), 2.8 mM of dNTPs (First Base Laboratories, Seri Kembangan, Malaysia) and 0.8 M of betaine (Sigma, St. Louis, MO, USA) was prepared. Briefly, a 25 µl LAMP reaction contained 12.5 µl of 2 × reaction mixture, 1 µl of *Bst* DNA polymerase (8 U), 1 µl of fluorescent dye, 5 pmol each of F3 and B3 primers, 40 pmol each of FIP and BIP primers, 20 pmol each of the LF and LB primers, and varying volumes of samples and nuclease-free water. A no-template control was included in each test. To determine the optimum reaction temperature, the reaction tubes were incubated at 61 °C, 63 °C, and 65 °C for 30 to 60 min either in a Loopamp Realtime Turbidimeter (Eiken Chemical, Taito-ku, Japan) or in a water bath. This was followed by an enzyme inactivation step at 80 °C for 5 min. Following the successful LAMP reaction, the use of a self-assembled LAMP reaction buffer was investigated.

### Interpretation of LAMP reaction outcome

The outcome of the LAMP reaction was interpreted by several approaches. In the Loopamp Realtime Turbidimeter (Eiken Chemical, Japan), positive amplification of LAMP was determined by the formation of a sigmoidal curve exceeding the 0.1 threshold value of absorbance read at 650 nm. The LAMP reaction tubes were also visually inspected for the calcein dye color change. LAMP amplification will result in a change in the color of calcein dye from orange to green. Alternatively, LAMP amplicons were also analyzed using 1.5% agarose gel and electrophoresed in 1 × TAE buffer (Sigma, St. Louis, MO, USA) at 90 V (8.4 V/cm) for 45 min. Next, the agarose gel was stained with 0.5 µg/ml of ethidium bromide and visualized with a gel documentation system (Bio-Rad, Hercules, CA, USA). A positively amplified LAMP reaction is usually characterized by the presence of a ladder-like band pattern on the agarose gel.

### Analytical sensitivity and specificity of the *secY* LAMP assay

Genomic DNA isolated from *L. interrogans* serovar Pomona was used to determine the sensitivity of the LAMP system based on the lower limit of detection. Ten-fold serial dilutions of the genomic DNA were performed, resulting in a range of concentrations from 100 ng to 0.01 ng. The diluted genomic DNAs were used as templates in the LAMP reaction performed under the optimized conditions. To determine the specificity of the *secY* LAMP assay, genomic DNAs isolated from 15 *Leptospira* reference strains and 9 non-*Leptospira* bacteria were used (Table 2) for this purpose. The LAMP reaction was performed using 300 ng of each respective genomic DNA. The LAMP reaction was conducted for 40 min to determine the sensitivity and specificity of the assay when tested on the genomic DNA of pure *Leptospira* cultures. However, in subsequent testing, the duration of the LAMP reaction was reduced to 30 min to improve its applicability as a rapid detection method.

### Validation with human and rat kidney specimens

All the collected specimens from humans and rats were subjected to the *secY* LAMP assay under the optimized condition. For comparison, PCR targeting the same region of *secY* as in LAMP were performed using the LAMP F3 and B3 primers on all of the specimens. The PCR assay was conducted in a 25-µl reaction consisting of 12.5 µl of 2 × GoTaq^®^ Green Master Mix (Promega, Madison, WI, USA), 5 pmol each of F3 and B3 primers, 3 µl DNA template and nuclease-free water. The PCR reaction was performed at 95 °C for 2 min followed by 35 cycles of denaturation at 95 °C for 30 s, annealing at 56 °C for 30 s, extension at 72 °C for 1 min, and a final extension at 72 °C for 5 min. The PCR products were then analyzed using agarose gel electrophoresis and randomly selected for purification and sequencing. The resultant sequences were then compared against the entries in the NCBI GenBank database using the BLAST tool (https://blast.ncbi.nlm.nih.gov/) to verify its identity.

**Table 2:** List of *Leptospira* reference strains and non-*Leptospira* bacteria used in specificity testing.

Species	Serovars
*Leptospira interrogans*	Pomona
*Leptospira interrogans*	Serawak
*Leptospira interrogans*	Canicola
*Leptospira interrogans*	Djasiman
*Leptospira interrogans*	Autumnalis
*Leptospira interrogans*	Australis
*Leptospira interrogans*	Pyrogenes
*Leptospira interrogans*	Lai
*Leptospira interrogans*	Copenhageni
*Leptospira interrogans*	Icterohaemorrhagiae
*Leptospira interrogans*	Bataviae
*Leptospira interrogans*	Hebdomadis
*Leptospira kirschneri*	Grippotyphosa
*Leptospira borgpetersenii*	Hardjo Bovis
*Leptospira biflexa*	Patoc
*Stenotrophomonas maltophilia*	–
*Pseudomonas aeruginosa*	–
*Acinetobacter baumanii*	–
*Escherichia coli* XL10G	–
*Streptococcus pyogenes* (clinical isolate)	–
Methicillin-resistant *Staphylococcus aureus* (MRSA, clinical isolate)	–
*Enterococcus faecalis* (clinical isolate)	–
*Enterococcus faecium* (clinical isolate)	–
*Clostridium difficile* (clinical isolate)	–

## Results

### Optimized *secY* LAMP assay

**Figure 1:**
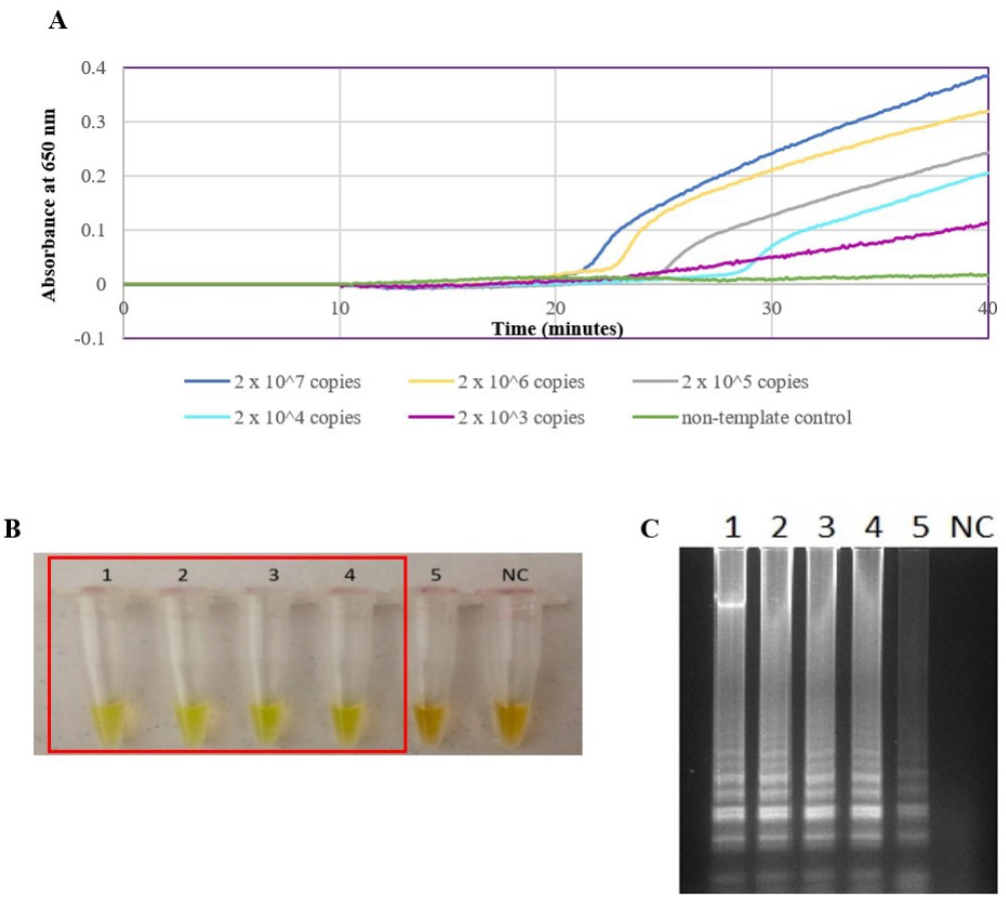
Sensitivity test of the LAMP assay using genomic DNA of *L. interrogans* serovar Pomona. The LAMP reaction was performed at 65 °C for 40 min. (A) Realtime turbidimetry result of the LAMP reaction. (B) Colorimetric observation based on calcein dye color change. (C) Agarose gel electrophoresis of LAMP reaction products. Tubes and lanes 1 to 5: 2 × 10^7^ (100 ng) to 2 × 10^3^ copies (0.01 ng) of genomic DNA per reaction, NC: no-template control. Red square box indicates reaction tubes with calcein color change.

**Figure 2:**
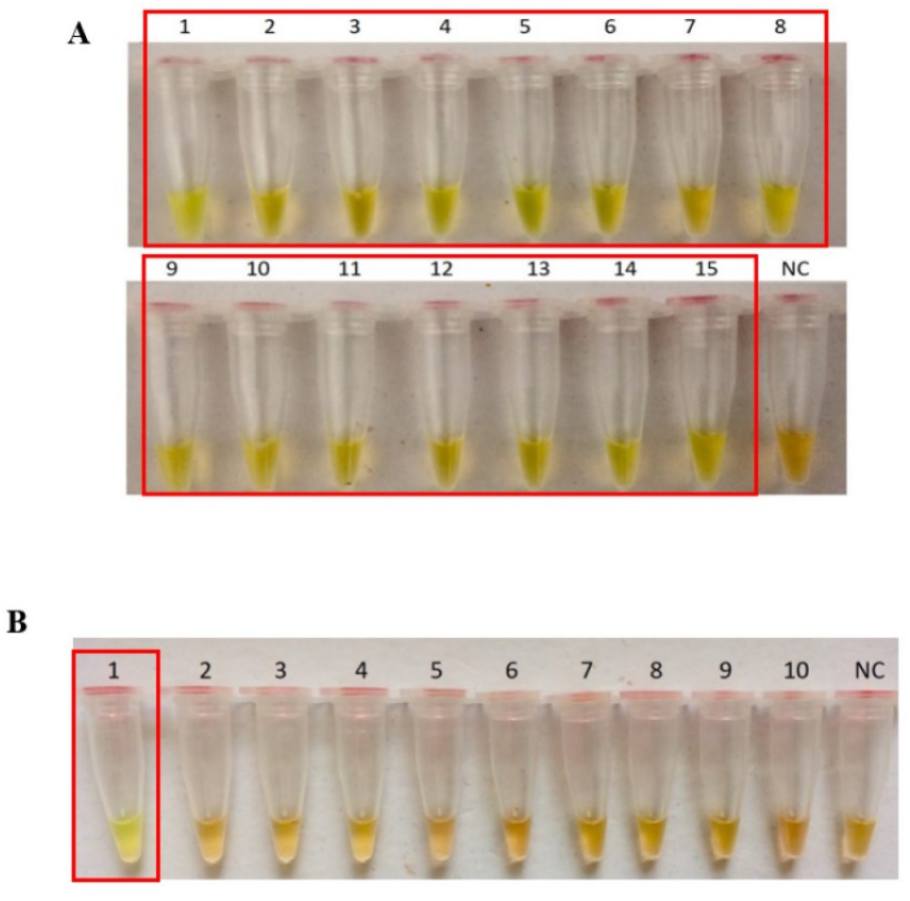
Specificity test of the LAMP assay based on colorimetric changes in calcein dye. The LAMP reaction was performed at 65 °C for 40 min using 300 ng of genomic DNA each. (A) Specificity test on different serovars of *Leptospira*. Tube 1: *L. interrogans* serovar Pomona, 2: *L. interrogans* serovar Serawak, 3: *L. interrogans* serovar Canicola, 4: *L. interrogans* serovar Djasiman, 5: *L. interrogans* serovar Autumnalis, 6: *L. interrogans* serovar Australis, 7: *L. interrogans* serovar Pyrogenes, 8: *L. interrogans* serovar Lai, 9: *L. interrogans* serovar Copenhageni, 10: *L. interrogans* serovar Icterohaemorrhagiae, 11: *L. interrogans* serovar Bataviae, 12: *L. interrogans* serovar Hebdomadis, 13: *L. kirschneri* serovar Grippotyphosa, 14: *L. borgpetersenii* serovar Hardjo Bovis 15: *L. biflexa* serovar Patoc, NC: no-template control. (B) Specificity test on non-leptospiral bacteria. Tube 1: *L. interrogans* Pomona, 2: *S. maltophillia* ATCC, 3: *P. aeruginosa*, 4: *A. baumannii* ATCC, 5: *E. coli* XL10G, 6: *S. pyogenes* S28, 7: MRSA, 8: *E. faecalis* 33420, 9: *E. faecium* 4867, 10: *C. difficile*, NC: no-template control. Red square box indicates reaction tubes with calcein color change.

The *secY* LAMP primers worked best at 65 °C with 0.4 M betaine and a final concentration of 8 mM MgSO_4_ in a 25 µl LAMP reaction. These conditions were used for all of the subsequent LAMP assays in this study. The sensitivity of the LAMP assay was assessed based on the lower limit of detection. The assay’s limit of detection for genomic DNA of *L. interrogans* serovar Pomona was 0.1 ng (equivalent to 2  × 10^4^ copies of genomic DNA) based on turbidimetry and observation of calcein color change at a reaction time of 40 min (Figs. 1A and 1B). Through agarose gel electrophoresis, a sensitivity of 0.01 ng was determined for the assay, though the ladder-like band pattern appeared fainter than the rest (Fig. 1C). Hence, for subsequent sensitivity analysis, the results of turbidimetry and color change observations were used for comparison. In terms of specificity, all 15 leptospiral DNA samples tested positive (Fig. 2A), and none of the non-leptospiral DNA samples showed amplification using the optimized *secY* LAMP assay (Fig. 2B).

**Figure 3:**
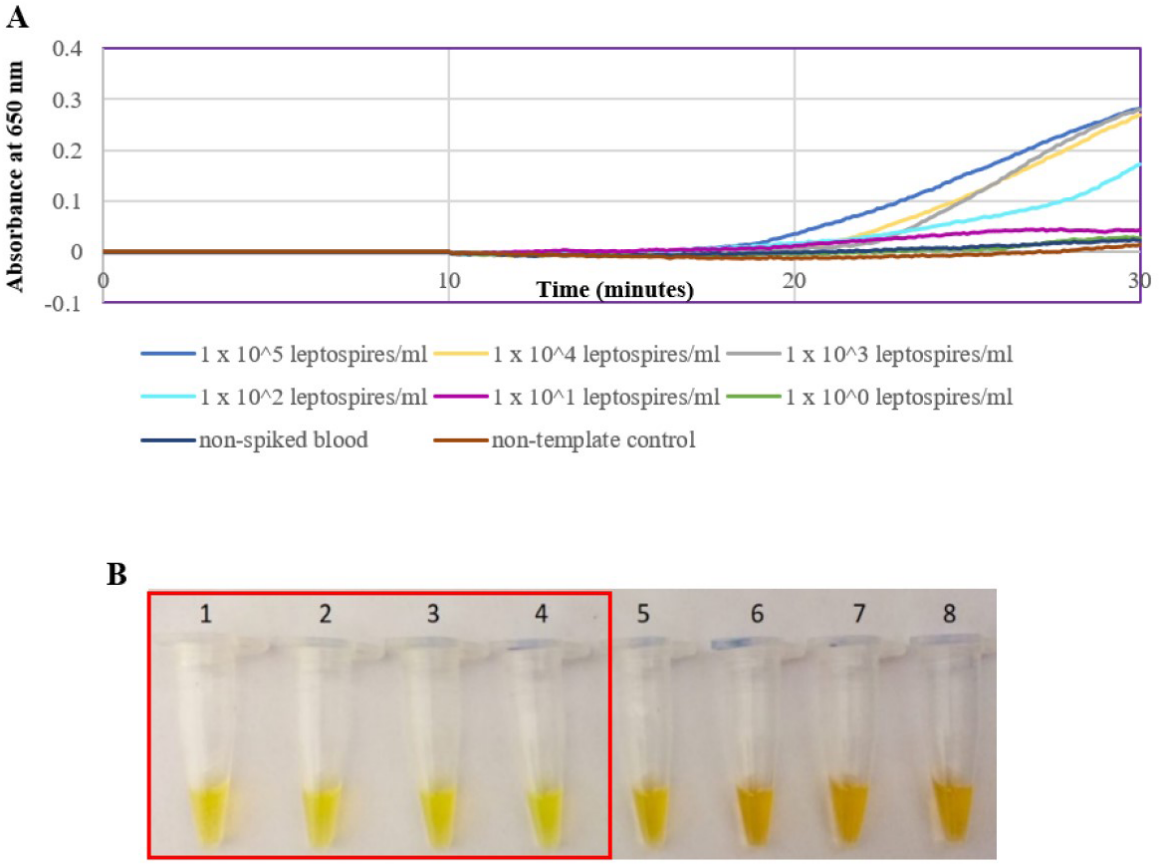
Sensitivity test of the LAMP assay using DNA isolated from spiked blood samples. The LAMP reaction was performed at 65 °C for 30 min. (A) Realtime turbidimetry result of the LAMP reaction. (B) Colorimetric observation based on calcein dye color change. Tubes 1 to 6: 1 × 10^5^ to 1 leptospires/ml, tube 7: non-spiked blood, tube 8: no-template control. Red square box indicates reaction tubes with calcein color change.

**Figure 4:**
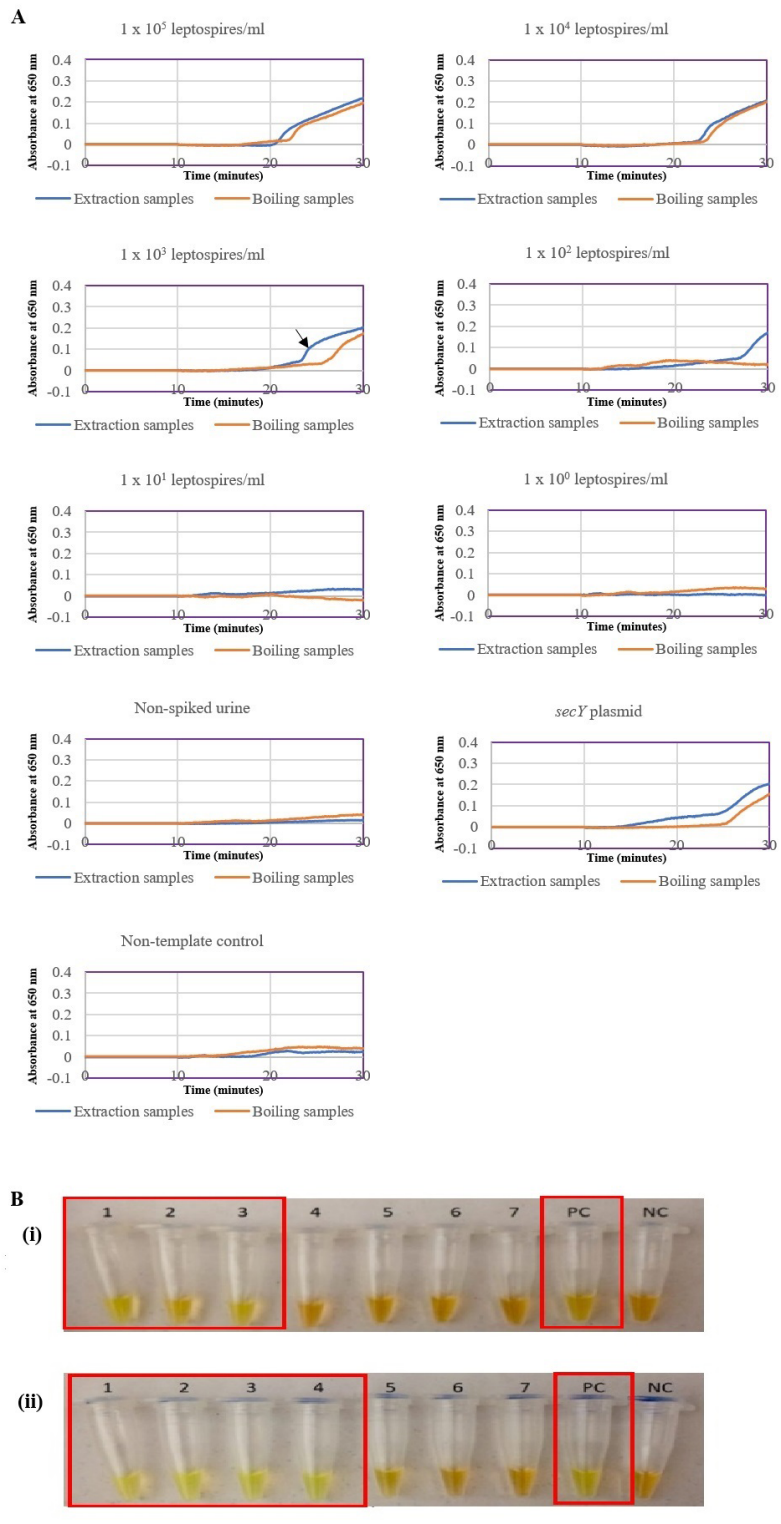
Sensitivity test of the LAMP assay using DNA isolated from spiked urine samples by extraction and the direct boiling method for comparison. The LAMP reaction was performed at 65 °C for 30 min. (A) Realtime turbidimetry result of the LAMP reaction. The black arrow on the graph of 1 × 10^3^ leptospires/ml indicates the shorter amplification time when tested on DNA extracted with a commercial extraction kit compared with DNA prepared by the direct lysis method. (B) (i) Colorimetric observation based on calcein dye color change. (i) Using DNA samples prepared by the direct lysis method (ii). Using DNA samples prepared by the column purification method. Tubes 1 to 6: 1 × 10^5^ to 1 leptospires/ml, Tube 7: non-spiked urine, PC, Positive control with secY plasmid; NC, no-template control. Red square box indicates reaction tubes with calcein color change.

The optimized *secY* LAMP assay was also tested on blood and urine samples artificially spiked with pure *Leptospira* culture of different dilutions. When tested on spiked blood samples, positive amplification was detected even at the dilution of 1 × 10^2^ leptospires/ml (Figs. 3A and 3B). No amplification was detected in non-spiked blood and the no-template control. DNA extracted from non-spiked blood was included as a negative control for the reaction to ensure that the donated blood was free of any leptospiral DNA. In the spiked urine specimens, the LAMP outcomes of two different DNA extraction approaches were studied and compared. As shown in Figs. 4A and 4B, when tested on DNA extracted through direct lysis method, the assay was able to detect 1 × 10^3^ leptospires/ml. However, faster amplification was observed as indicated by the arrow on graph 1 × 10^3^ leptospires/ml when tested on DNA extracted with a commercial extraction kit (Fig. 4A). This assay was capable of detecting down to 1 × 10^2^ leptospires/ml, comparable to that in spiked blood samples.

### Validation of the *secY* LAMP assay on suspected leptospirosis human specimens

A total of 69 blood samples were collected during admission; 28 samples (40.6%) were positive by LAMP, and 26 (37.7%) samples were positive by PCR. Meanwhile, for urine samples, 34 samples were collected during admission, and 16 (47.1%) and 14 (41.2%) were positive by LAMP and PCR, respectively. All of the discharge blood samples tested negative by both LAMP and PCR except for one (*n* = 1) sample, which was found to be positive by LAMP. On the other hand, LAMP and PCR conducted on discharge urine samples determined two (*n* = 2) samples to be positive despite being negative in the admission sample (Table 3). Sequencing of the randomly selected PCR products (Appendix A) revealed that the sequences had a 100% identity (align length 276 nt, Score 510, *E*-value 2e−143) to those of *L. interrogans* (Figs. S1–S6).

The presence of leptospiral DNA was detected in 45% of blood and urine samples collected. Collectively, 66% of the samples collected during admission were tested positive by the *secY* LAMP assay (Table 4).

### Validation of the *secY* LAMP assay on rat kidney specimens

A total of 88 rats were caught from suspected leptospirosis outbreak areas. Among these, 31 (35.2%) and 19 (21.6%) kidneys tested positive for the presence of leptospiral DNA detected by *secY* LAMP and PCR, respectively. On the other hand, 25 (40.3%) and 9 (14.5%) rat kidneys tested positive for leptospiral DNA from 62 rats caught from high-risk areas by LAMP and PCR, respectively (Table 5). The positive detection rate in the rat kidney was compared between LAMP and PCR and was higher in LAMP. In addition, all PCR-positive samples were also positive by LAMP. BLAST analysis also revealed that the sequenced PCR product (Appendix A) shared complete identity (align length 439 nt, score 811, *E*-value 3e−112) to those in *L. interrogans* (Fig. S7).

## Discussion

The approach of using the LAMP technique in this study was prompted by its simplicity and rapidity as compared with other DNA amplification techniques such as PCR and real time PCR. Previous studies had reported the development of LAMP system for the detection of leptospiral DNA targeting several *Leptospira*-specific genes, such as the *rrs* gene (Chen et al., 2015; Sonthayanon et al., 2011; Suwancharoen, Sittiwicheanwong & Wiratsudakul, 2016), *lipL32* (Chen et al., 2015), *lipL41* (Lin et al., 2009), and *ligB* (Ali et al., 2017). However, the development of a leptospirosis diagnostic test that only focuses on the detection of pathogenic strains may cause a nonresponsive result or false-negative outcome (Alizadeh et al., 2016). Therefore, the housekeeping *secY* gene was selected as the target gene to enable extensive detection of leptospiral DNA regardless of its pathogenicity, especially in clinical samples. In addition, a meta-analysis conducted on the diagnostic accuracy of various leptospiral genetic markers revealed that nucleic acid assays targeting the *secY* gene had better diagnostic accuracy measures and is one of the promising markers for detecting the pathogen (Lam, Low & Chee, 2020).

**Table 3:** Comparison of LAMP and PCR detection rates in suspected leptospirosis patient samples.

Sample type	Admission		Discharge	
	Blood	Urine	Blood	Urine
Number of samples tested	69	34	15	8
Positive by *secY* LAMP, n (%)	28 (40.6)	16 (47.1)	1 (6.7)	2 (25.0)
Positive by *secY* PCR, n (%)	26 (37.7)	14 (41.2)	0 (0)	2 (25.0)

**Table 4:** Proportion of secY LAMP positives among admission samples.

*secY* LAMP	Urine positive, %	Urine negative, %
Blood positive, %	24	21
Blood negative, %	21	33

**Table 5:** Comparison of LAMP and PCR detection rates in rat kidney samples.

Sample origin	Suspected leptospirosis outbreak area	High-risk area
Number of samples tested	88	62
Positive by *secY* LAMP, n (%)	31 (35.2)	25 (40.3)
Positive by *secY* PCR, n (%)	19 (21.6)	9 (14.5)

In this study, 2  × 10^4^ copies of genomic DNA (isolated from *L. interrogans* serovar Pomona) per reaction was found to be the lowest detection limit of the developed LAMP assay. Although Suwancharoen, Sittiwicheanwong & Wiratsudakul (2016) reported a lower detection limit of 10 and 100 copies using genomic DNA of serovars Tarasovi and Icterohaemorrhagiae, respectively (Suwancharoen, Sittiwicheanwong & Wiratsudakul, 2016), a longer incubation period of 90 min were required instead of a 40 min reaction as described in the current study, and a different gene was targeted. In sensitivity testing, three detection methods were used; turbidimetry, observation of the calcein color change, and agarose gel electrophoresis. Although a higher sensitivity was achieved by using electrophoresis, it was not the most practical approach, especially if the assay is intended for use in resource-limited settings, as multiple pieces of equipment and longer turnaround time are required. Meanwhile, a turbidimeter is indispensable if the choice of a detection method is based on the measurement of turbidity. Thus, a colorimetric endpoint detection method such as the calcein dye method is preferred as it is more translatable to the ultimate goal of point-of-care. The optimized LAMP system in this study showed no cross reactivity with a set of bacteria other than *Leptospira*. The specificity of the optimized LAMP system was also tested on 15 *Leptospira* strains, and all were successfully amplified, including a non-pathogenic species, *L. biflexa* serovar Patoc.

A spiking assay was performed to estimate the assay’s efficiency in a condition that closely resembles the clinical situation prior to evaluation on actual clinical samples. Here, a spiking assay was conducted on blood and urine samples. Whole blood samples were used instead of serum as DNA extracted from whole blood was reported to result in a higher detection rate (Bourhy et al., 2011) and that detection of leptospiral DNA in serum was reported to be less sensitive (Kositanont et al., 2007; Stoddard et al., 2009). The assay was able to detect down to 100 leptospires/ml, consistent with previous reports, although a different gene was targeted (Backstedt et al., 2015; Chen et al., 2015). In this study, two different approaches of DNA isolation from spiked urine samples were performed and compared. The direct lysis method was commonly used due to its simpler procedure for application in a resource-limited setting (Koizumi et al., 2012; Slack et al., 2006; Tubalinal et al., 2018). However, the findings here revealed a better rate of amplification and detection sensitivity of the LAMP reaction using DNA samples obtained from a commercial extraction kit, which may be attributed to the use of enzymes, a separation column and repeated washing steps in the protocol.

Both blood and urine samples from suspected leptospirosis patients were utilized for the validation of the current *secY* LAMP assay. The same region of *secY* gene was also targeted in conventional PCR to compare the detection rate of both nucleic acid amplification approaches. Based on the findings, LAMP demonstrated a higher number of positive detections compared to conventional PCR. A higher number of positive leptospiral DNA detection in clinical specimens through LAMP was also previously reported by Hsu et al. (2017) where 38.1% of the samples tested were positive by LAMP whereas only 4.8% of the samples were positive through conventional PCR (Hsu et al., 2017). As such, the difference of detection rate between the two LAMP assays could be attributed to the performance of the primers under a different operating temperature, which will affect the analytical sensitivity. Nevertheless, given the considerably high leptospiral detection rate in urine by both LAMP and PCR, it is suggested that apart from collecting blood as a diagnostic sample during the early days of infection, the collection of urine samples from suspected leptospirosis patients should also be considered and used for routine diagnosis and monitoring.

The epidemiology of human leptospirosis is complex and dynamic as it involves the interaction between the pathogen, animal reservoir, host, and environment. The coexistence between human and reservoir animals, particularly rats, in the same environment increases the likelihood of leptospirosis infection. Globally, leptospirosis has been associated with occupational activities; however, over the last few decades, climatic changes and natural disasters have been associated with changes in the epidemiology of human leptospirosis (Stern et al., 2010). While natural disasters may account for the increase in leptospirosis cases, the high prevalence of leptospires in rats has also translated to an increase in leptospirosis transmission to humans. Early detection of *Leptospira* spp. in carrier animals is crucial to prevent the spread of leptospirosis infection to other animals and humans. Thus, the *secY* LAMP assay developed in this study was also used to detect the presence of leptospiral DNA in rat kidney specimens.

The present study uses the same rat kidney samples from Azhari et al. (2018); henceforth, the term “suspected leptospirosis outbreak” was retained in this study (Azhari et al., 2018). Briefly, the rats were captured in an area where suspected leptospirosis cases had been reported. These areas include a recreational forest, an urban residential, and a semi-urban area in the Hulu Langat and Gombak districts, Selangor, Malaysia (Azhari et al., 2018). In addition, rat kidney samples from selected sites from wet markets, Selangor, Malaysia were included in this study. A study reported a high seroprevalence of leptospires among wet market workers (Rahman et al., 2018), which suggests that wet markets are potential locales of leptospirosis transmission. Thus, the term ‘high risk’ was used to represent wet markets in this study.

It is also worth noting that the leptospiral DNA detection rate was higher in LAMP than in PCR for all the samples. The difference was even more striking when these methods were tested on rat kidney samples. This could be explained by the presence of inhibitors specific to PCR. Inhibitory substances, such as collagen, myoglobin, and heparin, which were found in rat tissue samples, may be co-extracted with the DNA during the sample extraction process, which eventually reduces the amplification efficiency of PCR (Bessetti, 2007). Aside from inhibitory substances, the choice of DNA polymerase also plays a crucial role in the sensitivity of the test. *Taq* DNA polymerase, which is used in PCR, can be inhibited by biological materials present in the test samples (Abdullahi et al., 2015; Burkardt, 2000). However, this problem is not applicable in LAMP, as the assay uses *Bst* DNA polymerase, which is less susceptible to inhibitory substances (Rohatensky et al., 2018). Other studies have shown better tolerance to inhibitor substances in LAMP than in PCR, regardless of the sample types used (Li et al., 2012; Njiru et al., 2008; Yeh, Shoemaker & Klesius, 2005).

There are some limitations to the present study. Firstly, detection of *Leptospira* using LAMP was compared only with PCR method. MAT which is regarded as the gold standard for diagnosis of leptospirosis was not performed because of its inability to detect leptospirosis in early stages of infection (Niloofa et al., 2015). Secondly, although the present study demonstrated the detection of *secY* gene of *Leptospira*, however other genetic markers such as *lipL32, rrs*, *lipL41* and *flaB* could be included as different targeted gene may impact on the sensitivity and specificity of the test (Lam, Low & Chee, 2020). Further studies into other different genes of *Leptospira* are warranted.

## Conclusion

Collectively, we have devised a robust, rapid, sensitive, and specific nucleic acid test based on LAMP to detect the presence of *Leptospira* spp. by targeting the *secY* gene. The assay was validated on both human specimens and rat kidney specimens, which expands its applicable use in healthcare as well as environmental surveillance of the disease. The developed LAMP assay can serve as a promising alternative tool to tackle the diagnostic problem of the disease and to control the spread of the disease with better surveillance of the vectors.

## Additional Information and Declarations

### Data Availability

The following information was supplied regarding data availability: The raw measurements are available in the Supplementary Files. The sequences are available at NCBI GenBank: OK362289, OK362290, OK362291, OK362292, OK362293, OK362294, OK317697.

## Supplemental Information

Supplemental Information 1 Sequencing and BLAST analysis of PCR reaction products from clinical blood samples (Sample 011)

Supplemental Information 2 Sequencing and BLAST analysis of PCR reaction products from clinical blood samples (Sample 058)

Supplemental Information 3 Sequencing and BLAST analysis of PCR reaction products from clinical urine samples (Sample 001)

Supplemental Information 4 Sequencing and BLAST analysis of PCR reaction products from clinical urine samples (Sample 005)

Supplemental Information 5 Sequencing and BLAST analysis of PCR reaction products from clinical urine samples (Sample 019)

Supplemental Information 6 Sequencing and BLAST analysis of PCR reaction products from rat kidney samples (Sample 155)

Supplemental Information 7 PCR for Clinical and animal samples PCR result for blood urine and rat kidney samples.

Supplemental Information 8 secY Primers Alignment Map

Supplemental Information 9 Raw Data for Comparison of LAMP and PCR detection rates in suspected leptospirosis patient samples (Table 3)

Supplemental Information 10 Raw Data Proportion of secY LAMP positives among admission samples (Table 4)

Supplemental Information 11 Raw data for detection rates of rat kidney samples (Table 5)

Supplemental Information 12 Arrive Guideline Checklist for Animal Studies

Supplemental Information 13 Raw sequence data F3 (Fig. S1) The file can be viewed using Geneious Prime software.

Supplemental Information 14 Raw sequence data B3 (Fig. S1) The file can be viewed using Geneious Prime software.

Supplemental Information 15 Raw sequence data F3 (Fig. S2) The file can be viewed using Geneious Prime software.

Supplemental Information 16 Raw sequence data B3 (Fig. S2) The file can be viewed using Geneious Prime software.

Supplemental Information 17 Raw sequence data F3 (Fig. S3) The file can be viewed using Geneious Prime software.

Supplemental Information 18 Raw sequence data B3 (Fig. S3) The file can be viewed using Geneious Prime software.

Supplemental Information 19 Raw sequence data F3 (Fig. S4)

Supplemental Information 20 Raw sequence data B3 (Fig. S4)

Supplemental Information 21 Raw sequence data F3 (Fig. S5) The file can be viewed using Geneious Prime software.

Supplemental Information 22 Raw sequence data B3 (Fig. S5) The file can be viewed using Geneious Prime software.

Supplemental Information 23 Raw sequence data F3 (Fig. S6) The file can be viewed using Geneious Prime software.

Supplemental Information 24 Raw sequence data B3 (Fig. S6) The file can be viewed using Geneious Prime software.

Supplemental Information 25 Raw sequence data F3 (Fig. S7) The file can be viewed using Geneious Prime software.

Supplemental Information 26 Raw sequence data B3 (Fig. S7) The file can be viewed using Geneious Prime software.

## References

[ref-1] Abdullahi UF, Naim R, Taib WRW, Saleh A, azu AMu, Aliyu S, Baig AA (2015). Loop-mediated isothermal amplification (LAMP), an innovation in gene amplification: bridging the gap in molecular diagnostics; a review. Indian Journal of Science and Technology.

[ref-2] Adler B, Adler B (2015). History of leptospirosis and leptospira. Leptospira and leptospirosis.

[ref-3] Adler B, de la Peña Moctezuma A (2010). Leptospira and leptospirosis. Veterinary Microbiology.

[ref-4] Ahmad SN, Shah S, Ahmad FM (2005). Laboratory diagnosis of leptospirosis. Journal of Postgraduate Medicine.

[ref-5] Ahmed A, Engelberts MF, Boer KR, Ahmed N, Hartskeerl RA (2009). Development and validation of a real-time PCR for detection of pathogenic leptospira species in clinical materials. PLOS ONE.

[ref-6] Ahmed A, Linden Hvander, Hartskeerl RA (2014). Development of a recombinase polymerase amplification assay for the detection of pathogenic Leptospira. International Journal of Environmental Research and Public Health.

[ref-7] Ali SA, Kaur G, Boby N, Sabarinath T, Solanki K, Pal D, Chaudhuri P (2017). Rapid and visual detection of Leptospira in urine by LigB-LAMP assay with pre-addition of dye. Molecular and Cellular Probes.

[ref-8] Alizadeh S, Javadi A, Alizadeh S, Najafipour R, Naserpour T (2016). Simultaneous detection of pathogenic and saprophyte leptospira in human plasma by multiplex taqman real time PCR. Biotechnology and Health Sciences.

[ref-9] Aslan O (2016). Leptospirosis; diagnosis, treatment and prevention: a review. British Microbiology Research Journal.

[ref-10] Azhari NN, Ramli SNA, Joseph N, Philip N, Mustapha NF, Ishak SN, Mohd-Taib FS, Nor SMd, Yusof MA, Sah SAMohd, Desa MNBMohd, Bashiru G, Zeppelini CG, Costa F, Sekawi Z, Neela VK (2018). Molecular characterization of pathogenic Leptospira sp. in small mammals captured from the human leptospirosis suspected areas of Selangor state, Malaysia. Acta Tropics.

[ref-11] Backstedt BT, Buyuktanir O, Lindow J, Wunde. Jr EA, Reis MG, Usmani-Brown S, Ledizet M, Ko A, Pal U (2015). Efficient detection of pathogenic leptospires using 16S ribosomal RNA. PLOS ONE.

[ref-12] Bahtiar Affendy N, Mohd Desa MN, Amran F, Sekawi Z, Masri SN (2020). Isolation and molecular characterization of Leptospira interrogans and Leptospira borgpetersenii from small mammals in Selangor wet markets. International Journal of Infectious Diseases.

[ref-13] Bedir O, Kilic A, Atabek E, Kuskucu AM, Turhan V, Basustaoglu AC (2010). Simultaneous detection and differentiation of pathogenic and nonpathogenic Leptospira spp. by multiplex real-time PCR (TaqMan) assay. Polish Journal of Microbiology.

[ref-14] Bessetti J (2007). An introduction to PCR inhibitors. Profiles DNA.

[ref-15] Bourhy P, Bremont S, Zinini F, Giry C, Picardeau M (2011). Comparison of real-time PCR assays for detection of pathogenic Leptospira spp. in blood and identification of variations in target sequences. Journal of Clinical Microbiology.

[ref-16] Burkardt HJ (2000). Standardization and quality control of PCR analyses. Clinical Chemistry and Laboratory Medicine.

[ref-17] Chen HW, Weissenberger G, Atkins E, Chao CC, Suputtamongkol Y, Ching WM (2015). Highly sensitive loop-mediated isothermal amplification for the detection of leptospira. International Journal of Bacteriology.

[ref-18] Fouts DE, Matthias MA, Adhikarla H, Adler B, Amorim-Santos L, Berg DE, Bulach D, Buschiazzo A, Chang YF, Galloway RL, Haake DA, Haft DH, Hartskeerl R, Ko AI, Levett PN, Matsunaga J, Mechaly AE, Monk JM, Nascimento AL, Nelson KE, Palsson B, Peacock SJ, Picardeau M, Ricaldi JN, Thaipandungpanit J, Wunde Jr EA, Yang XF, Zhang JJ, Vinetz JM (2016). What makes a bacterial species pathogenic? Comparative genomic analysis of the genus leptospira. PLOS Neglected Tropical Diseases.

[ref-19] Hsu YH, Chou SJ, Chang CC, Pan MJ, Yang WC, Lin CF, Chan KW (2017). Development and validation of a new loop-mediated isothermal amplification for detection of pathogenic Leptospira species in clinical materials. The Journal of Microbiological Methods.

[ref-20] King AM, Bartpho T, Sermswan RW, Bulach DM, Eshghi A, Picardeau M, Adler B, Murray GL (2013). Leptospiral outer membrane protein LipL41 is not essential for acute leptospirosis but requires a small chaperone protein, lep, for stable expression. Infection and Immunity.

[ref-21] Koizumi N, Nakajima C, Harunari T, Tanikawa T, Tokiwa T, Uchimura E, Furuya T, Mingala CN, Villanueva MA, Ohnishi M, Suzuki Y (2012). A new loop-mediated isothermal amplification method for rapid, simple, and sensitive detection of Leptospira spp. in urine. Journal of Clinical Microbiology.

[ref-22] Kositanont U, Rugsasuk S, Leelaporn A, Phulsuksombati D, Tantitanawat S, Naigowit P (2007). Detection and differentiation between pathogenic and saprophytic Leptospira spp. by multiplex polymerase chain reaction. Diagnostic Microbiology and Infectious Disease.

[ref-23] Lam JY, Low GK, Chee HY (2020). Diagnostic accuracy of genetic markers and nucleic acid techniques for the detection of Leptospira in clinical samples: a meta-analysis. PLOS Neglected Tropical Diseases.

[ref-24] Lee P-Y, Wong Y-P, Othman S, Chee H-Y (2021). Room-temperature stable loop-mediated isothermal amplification (LAMP) reagents to detect leptospiral DNA. Asian Biomedicine.

[ref-25] Levett PN (2001). Leptospirosis. Clinical Microbiology Reviews.

[ref-26] Li X, Liu W, Wang J, Zou D, Wang X, Yang Z, Yin Z, Cui Q, Shang W, Li H, Wei X, Cui J, Wang Z, Huang L, Yuan J (2012). Rapid detection of Trichinella spiralis larvae in muscles by loop-mediated isothermal amplification. International Journal for Parasitology.

[ref-27] Lin X, Chen Y, Lu Y, Yan J, Yan J (2009). Application of a loop-mediated isothermal amplification method for the detection of pathogenic Leptospira. Diagnostic Microbiology and Infectious Disease.

[ref-28] Matsunaga J, Barocchi MA, Croda J, Young TA, Sanchez Y, Siqueira I, Bolin CA, Reis MG, Riley LW, Haake DA, Ko AI (2003). Pathogenic Leptospira species express surface-exposed proteins belonging to the bacterial immunoglobulin superfamily. Molecular Microbiology.

[ref-29] McBride AJ, Athanazio DA, Reis MG, Ko AI (2005). Leptospirosis. Current Opinion in Infectious Diseases.

[ref-30] Niloofa R, Fernando N, de Silva NL, Karunanayake L, Wickramasinghe H, Dikmadugoda N, Premawansa G, Wickramasinghe R, de Silva HJ, Premawansa S, Rajapakse S, Handunnetti S (2015). Diagnosis of Leptospirosis: comparison between microscopic agglutination test, IgM-ELISA and IgM rapid immunochromatography test. PLOS ONE.

[ref-31] Njiru ZK, Mikosza AS, Matovu E, Enyaru JC, Ouma JO, Kibona SN, Thompson RC, Ndung’u JM (2008). African trypanosomiasis: sensitive and rapid detection of the sub-genus Trypanozoon by loop-mediated isothermal amplification (LAMP) of parasite DNA. International Journal for Parasitology.

[ref-32] Notomi T, Okayama H, Masubuchi H, Yonekawa T, Watanabe K, Amino N, Hase T (2000). Loop-mediated isothermal amplification of DNA. Nucleic Acids Research.

[ref-33] Nurul Najian AB, Engku Nur Syafirah EA, Ismail N, Mohamed M, Yean CY (2016). Development of multiplex loop mediated isothermal amplification (m-LAMP) label-based gold nanoparticles lateral flow dipstick biosensor for detection of pathogenic Leptospira. Analytica Chimica Acta.

[ref-34] Rahman M, Hairon SM, Hamat RA, Jamaluddin T, Shafei MN, Idris N, Osman M, Sukeri S, Wahab ZA, Mohammad W, Idris Z, Daud A (2018). Seroprevalence and distribution of leptospirosis serovars among wet market workers in northeastern, Malaysia: a cross sectional study. BMC Infectious Diseases.

[ref-35] Richtzenhain LJ, Cortez A, Heinemann MB, Soares RM, Sakamoto SM, Vasconcellos SA, Higa ZM, Scarcelli E, Genovez ME (2002). A multiplex PCR for the detection of Brucella spp. and Leptospira spp. DNA from aborted bovine fetuses. Veterinary Microbiology.

[ref-36] Rodríguez I, Rodríguez I, Fernández C, Rodríguez JE, Cantillo J (2013). Detection of leptospires from infected urine and tissue samples in vitro by modified Fontana silver stain. Jornal Brasileiro de Patologia e Medicina Laboratorial.

[ref-37] Rohatensky MG, Livingstone DM, Mintchev P, Barnes HK, Nakoneshny SC, Demetrick DJ, Dort JC, van Marle G (2018). Assessing the performance of a Loop Mediated Isothermal Amplification (LAMP) assay for the detection and subtyping of high-risk suptypes of Human Papilloma Virus (HPV) for Oropharyngeal Squamous Cell Carcinoma (OPSCC) without DNA purification. BMC Cancer.

[ref-38] Saharan P, Dhingolia S, Khatri P, Duhan J, Gahlawat S (2014). Loop-mediated isothermal amplification (LAMP) based detection of bacteria: a review. African Journal of Biotechnology.

[ref-39] Salgado M, Otto B, Moroni M, Sandoval E, Reinhardt G, Boqvist S, Encina C, Muñoz Zanzi C (2015). Isolation of Leptospira interrogans serovar Hardjoprajitno from a calf with clinical leptospirosis in Chile. BMC Veterinary Research.

[ref-40] Sengupta M, Prabhakar AK, Satyendra S, Thambu D, Abraham OC, Balaji V, Chen HW, Chao CC, Ching WM, Prakash JA (2017). Utility of loop-mediated isothermal amplification assay, polymerase chain reaction, and ELISA for diagnosis of leptospirosis in South Indian patients. Journal of Global Infectious Diseases.

[ref-41] Slack AT, Symonds ML, Dohnt MF, Smythe LD (2006). Identification of pathogenic Leptospira species by conventional or real-time PCR and sequencing of the DNA gyrase subunit B encoding gene. BMC Microbiology.

[ref-42] Sonthayanon P, Chierakul W, Wuthiekanun V, Thaipadungpanit J, Kalambaheti T, Boonsilp S, Amornchai P, Smythe LD, Limmathurotsakul D, Day NP, Peacock SJ (2011). Accuracy of loop-mediated isothermal amplification for diagnosis of human leptospirosis in Thailand. The American Society of Tropical Medicine and Hygiene.

[ref-43] Stern EJ, Galloway R, Shadomy SV, Wannemuehler K, Atrubin D, Blackmore C, Wofford T, Wilkins PP, Ari MD, Harris L, Clark TA (2010). Outbreak of leptospirosis among Adventure Race participants in Florida, 2005. Clinical Infectious Diseases.

[ref-44] Stoddard RA, Gee JE, Wilkins PP, McCaustland K, Hoffmaster AR (2009). Detection of pathogenic Leptospira spp. through TaqMan polymerase chain reaction targeting the LipL32 gene. Diagnostic Microbiology and Infectious Disease.

[ref-45] Suwancharoen D, Sittiwicheanwong B, Wiratsudakul A (2016). Evaluation of loop-mediated isothermal amplification method (LAMP) for pathogenic Leptospira spp. detection with leptospires isolation and real-time PCR. The Journal of Veterinary Medical Science.

[ref-46] Thibeaux R, Girault D, Bierque E, Soupé-Gilbert M-E, Rettinger A, Douyère A, Meyer M, Iraola G, Picardeau M, Goarant C (2018). Biodiversity of environmental leptospira: improving identification and revisiting the diagnosis. Frontiers in Microbiology.

[ref-47] Toyokawa T, Ohnishi M, Koizumi N (2011). Diagnosis of acute leptospirosis. Expert Review of Anti-infective Therapy.

[ref-48] Tubalinal GAS, Balbin MM, Villanueva MA, Domingo CYJ, Mingala CN (2018). Evaluation of LAMP for detection and/or screening of Leptospira spp. infection among domestic animals in the Philippines. Journal of Advanced Veterinary and Animal Research.

[ref-49] Xiao D, Zhang C, Zhang H, Li X, Jiang X, Zhang J (2015). A novel approach for differentiating pathogenic and non-pathogenic Leptospira based on molecular fingerprinting. Journal of Proteomics.

[ref-50] Yeh HY, Shoemaker CA, Klesius PH (2005). Evaluation of a loop-mediated isothermal amplification method for rapid detection of channel catfish Ictalurus punctatus important bacterial pathogen Edwardsiella ictaluri. The Journal of Microbiological Methods.

[ref-51] Zenebe T, Abdi R, Keskes S (2013). Global epidemiological overview of leptospirosis. International Journal of Microbiology.

[ref-52] Zuerner RL, Hartskeerl RA, vande Kemp H, Bal AE (2000). Characterization of the Leptospira interrogans S10-spc- *α* operon. FEMS Microbiology Letters.

