# Supplementary material for: A versatile isothermal amplification assay for the detection of leptospires from various sample types
Source: PeerJ. 2022 Mar 10;10:e12850. doi: 10.7717/peerj.12850 (PMC8918162; doi:10.7717/peerj.12850)
Supplement: Supplemental Information 3 [file peerj-10-12850-s003.pdf]

Sequences producing significant alignments

Download | New Select columns | Show 10

select all 10 sequences selected

GenBank Graphics Distance tree of results New MSA Viewer

	Description	Scientific Name	Max Score	Total Score	Query Cover	E value	Per. Ident	Acc. Len	Accession
☑	Leptospira interrogans serovar Canicola strain 611 chromosome 1, complete sequence	Leptospira interrogans serovar C...	510	510	100%	2e-143	100.00%	4255595	CP044513.1
☑	Leptospira interrogans serovar Canicola strain LJ178 chromosome 1, complete sequence	Leptospira interrogans serovar C...	510	510	100%	2e-143	100.00%	4259066	CP044509.1
☑	Leptospira interrogans serovar Canicola strain 114 chromosome I sequence	Leptospira interrogans serovar C...	510	510	100%	2e-143	100.00%	4218946	CP022883.1
☑	Leptospira interrogans serovar Linhai str. 56609 chromosome 1, complete sequence	Leptospira interrogans serovar Li...	510	510	100%	2e-143	100.00%	4331770	CP006723.1
☑	Leptospira interrogans serovar Gem strain Simon secY gene, partial sequence	Leptospira interrogans serovar G...	510	510	100%	2e-143	100.00%	1408	EU358039.1
☑	Leptospira interrogans serovar Pomona strain LT 1026 SecY (secY) gene, partial cds	Leptospira interrogans serovar P...	510	510	100%	2e-143	100.00%	1375	EU358017.1
☑	Leptospira interrogans serovar Monjakov SecY (secY) gene, partial cds	Leptospira interrogans serovar M...	510	510	100%	2e-143	100.00%	1386	EU358014.1
☑	Leptospira interrogans serovar Pomona SecY (secY) gene, partial cds	Leptospira interrogans serovar P...	510	510	100%	2e-143	100.00%	1382	EU358013.1
☑	Leptospira interrogans serovar Medanensis strain Hond HC SecY (secY) gene, partial cds	Leptospira interrogans serovar M...	510	510	100%	2e-143	100.00%	1386	EU357984.1
☑	Leptospira interrogans serovar Bratislava strain Jez Bratislava SecY (secY) gene, partial cds	Leptospira interrogans serovar Br...	510	510	100%	2e-143	100.00%	1386	EU357939.1

Download GenBank Graphics

Leptospira interrogans serovar Gem strain Simon secY gene, partial sequence

Sequence ID: EU358039.1 **Length:** 1408 **Number of Matches:** 1

Range 1: 163 to 438 GenBank Graphics ▼ Next Match ▲ Previous Match

Score	Expect	Identities	Gaps	Strand
510 bits(276)	2e-143	276/276(100%)	0/276(0%)	Plus/Plus

```
Query  1    CTTGTTCCTGCCCTTCaaaaacttcaaaaagaaggagaagaaggaagaaaaaaaaTTGGT  60
            ||||||||||||||||||||||||||||||||||||||||||||||||||||||||||||
Sbjct  163  CTTGTTCCTGCCCTTCAAAAACTTCAAAAAGAAGGAGAAGAAGGAAGAAAAAAAAATTGGT  222

Query  61   CAATATACTAAGTATGGAACCGTAATTCTTTGTGCGATTCAATCTTTAGCAGTGATCCAA  120
            ||||||||||||||||||||||||||||||||||||||||||||||||||||||||||||
Sbjct  223  CAATATACTAAGTATGGAACCGTAATTCTTTGTGCGATTCAATCTTTAGCAGTGATCCAA  282

Query  121  CTCGCAAAGGGCTGGTCTACCGGCACGGAACTTGAGCCTGCGCGTTACCCGGGCTTAATC  180
            ||||||||||||||||||||||||||||||||||||||||||||||||||||||||||||
Sbjct  283  CTCGCAAAGGGCTGGTCTACCGGCACGGAACTTGAGCCTGCGCGTTACCCGGGCTTAATC  342

Query  181  AATTCTTCTGTTGTTCCTTATTTTTATTTAATCGGAATCTTATCCATTACTACCGGAACC  240
            ||||||||||||||||||||||||||||||||||||||||||||||||||||||||||||
Sbjct  343  AATTCTTCTGTTGTTCCTTATTTTTATTTAATCGGAATCTTATCCATTACTACCGGAACC  402

Query  241  GTTCTTCTCATTTGGTTAGGAGAACAGATCACCGAA  276
            ||||||||||||||||||||||||||||||||||||
Sbjct  403  GTTCTTCTCATTTGGTTAGGAGAACAGATCACCGAA  438
```

Figure S4. Sequencing and BLAST analysis of PCR reaction products from clinical urine samples (Sample 001).
