# Supplementary material for: A versatile isothermal amplification assay for the detection of leptospires from various sample types
Source: PeerJ. 2022 Mar 10;10:e12850. doi: 10.7717/peerj.12850 (PMC8918162; doi:10.7717/peerj.12850)
Supplement: Supplemental Information 6 [file peerj-10-12850-s006.pdf]

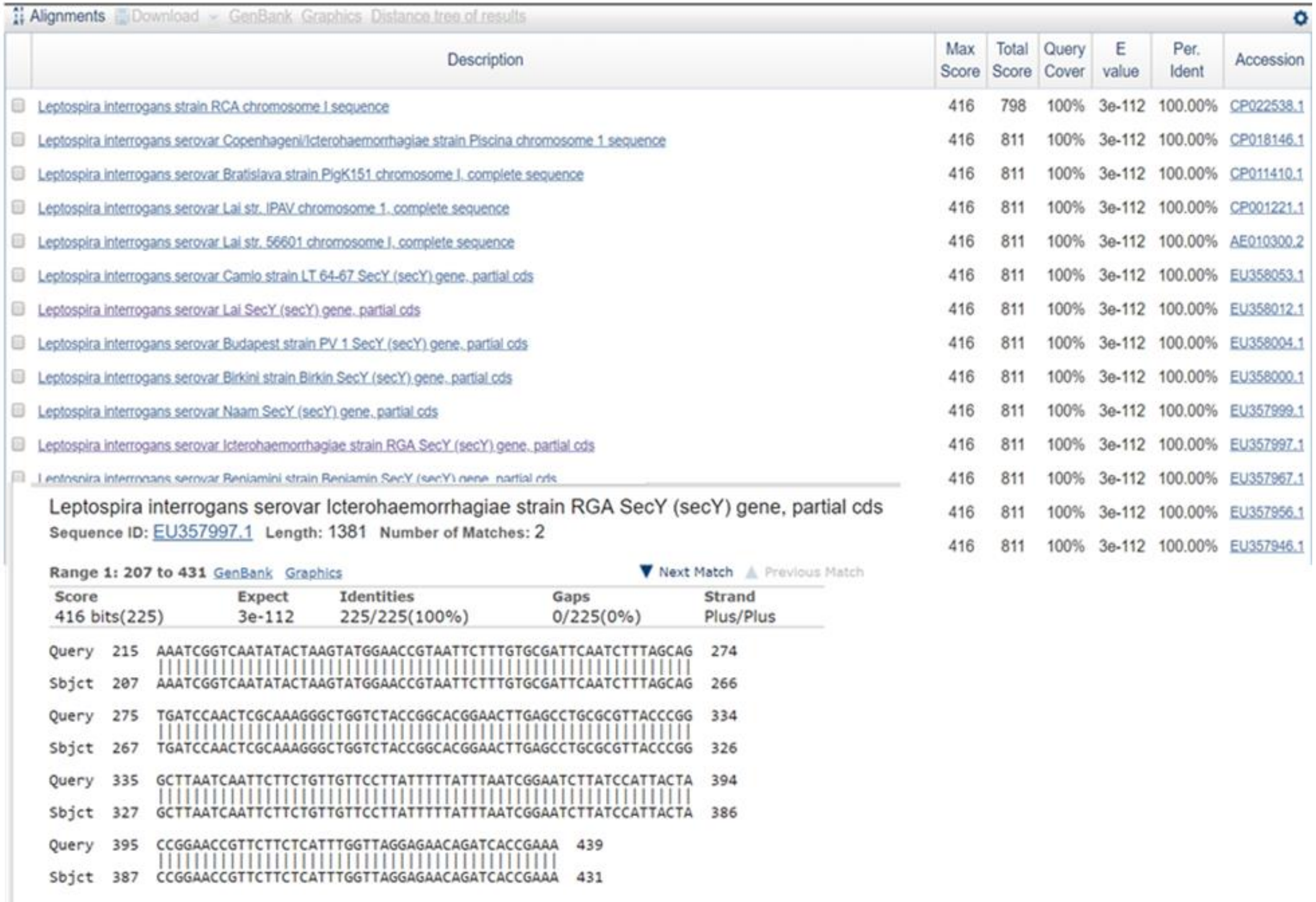

Alignments Download GenBank Graphics Distance tree of results

Description	Max Score	Total Score	Query Cover	E value	Per. Ident	Accession
Leptospira interrogans strain RCA chromosome I sequence	416	798	100%	3e-112	100.00%	CP022538.1
Leptospira interrogans serovar Copenhageni/Icterohaemorrhagiae strain Piscina chromosome 1 sequence	416	811	100%	3e-112	100.00%	CP018146.1
Leptospira interrogans serovar Bratislava strain PigK151 chromosome I, complete sequence	416	811	100%	3e-112	100.00%	CP011410.1
Leptospira interrogans serovar Lai str. IPAV chromosome 1, complete sequence	416	811	100%	3e-112	100.00%	CP001221.1
Leptospira interrogans serovar Lai str. 56601 chromosome I, complete sequence	416	811	100%	3e-112	100.00%	AE010300.2
Leptospira interrogans serovar Canicola strain LT 64-67 SecY (secY) gene, partial cds	416	811	100%	3e-112	100.00%	EU358053.1
Leptospira interrogans serovar Lai SecY (secY) gene, partial cds	416	811	100%	3e-112	100.00%	EU358012.1
Leptospira interrogans serovar Budapest strain PV 1 SecY (secY) gene, partial cds	416	811	100%	3e-112	100.00%	EU358004.1
Leptospira interrogans serovar Birkini strain Birkin SecY (secY) gene, partial cds	416	811	100%	3e-112	100.00%	EU358000.1
Leptospira interrogans serovar Naam SecY (secY) gene, partial cds	416	811	100%	3e-112	100.00%	EU357999.1
Leptospira interrogans serovar Icterohaemorrhagiae strain RGA SecY (secY) gene, partial cds	416	811	100%	3e-112	100.00%	EU357997.1
Leptospira interrogans serovar Benjamini strain Benjamin SecY (secY) gene, partial cds	416	811	100%	3e-112	100.00%	EU357967.1
[illegible]	416	811	100%	3e-112	100.00%	EU357956.1
[illegible]	416	811	100%	3e-112	100.00%	EU357946.1

Leptospira interrogans serovar Icterohaemorrhagiae strain RGA SecY (secY) gene, partial cds

Sequence ID: EU357997.1 Length: 1381 Number of Matches: 2

Range 1: 207 to 431 GenBank Graphics Next Match Previous Match

Score	Expect	Identities	Gaps	Strand
416 bits(225)	3e-112	225/225(100%)	0/225(0%)	Plus/Plus

```
Query  215  AAATCGGTCAATATACTAAGTATGGAACCGTAATTCTTTGTGCGATTCAATCTTTAGCAG  274
            ||||||||||||||||||||||||||||||||||||||||||||||||||||||||||||
Sbjct  207  AAATCGGTCAATATACTAAGTATGGAACCGTAATTCTTTGTGCGATTCAATCTTTAGCAG  266

Query  275  TGATCCAACTCGCAAAGGGCTGGTCTACCGGCACGGAACTTGAGCCTGCGCGTTACCCGG  334
            ||||||||||||||||||||||||||||||||||||||||||||||||||||||||||||
Sbjct  267  TGATCCAACTCGCAAAGGGCTGGTCTACCGGCACGGAACTTGAGCCTGCGCGTTACCCGG  326

Query  335  GCTTAATCAATTCTTCTGTTGTTCCTTATTTTTATTTAATCGGAATCTTATCCATTACTA  394
            ||||||||||||||||||||||||||||||||||||||||||||||||||||||||||||
Sbjct  327  GCTTAATCAATTCTTCTGTTGTTCCTTATTTTTATTTAATCGGAATCTTATCCATTACTA  386

Query  395  CCGGAACCGTTCTTCTCATTTGGTTAGGAGAACAGATCACCGAAA  439
            |||||||||||||||||||||||||||||||||||||||||||||
Sbjct  387  CCGGAACCGTTCTTCTCATTTGGTTAGGAGAACAGATCACCGAAA  431
```

Figure S7. Sequencing and BLAST analysis of PCR reaction products from rat kidney samples (Sample 454).
