# Supplementary material for: A versatile isothermal amplification assay for the detection of leptospires from various sample types
Source: PeerJ. 2022 Mar 10;10:e12850. doi: 10.7717/peerj.12850 (PMC8918162; doi:10.7717/peerj.12850)
Supplement: Supplemental Information 7 — PCR result for blood urine and rat kidney samples. [file peerj-10-12850-s007.docx]

1. PCR of blood clinical samples

**
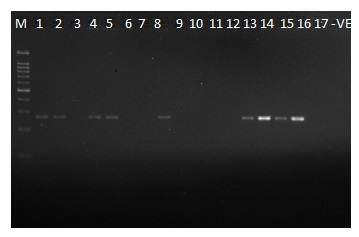
**

M: GenDirex 100 bp DNA ladder

Lane 1 – 17: Clinical samples of blood

-VE: Non-template control

PCR amplified product : 276 bp size

200

300

100

1. PCR of urine clinical samples


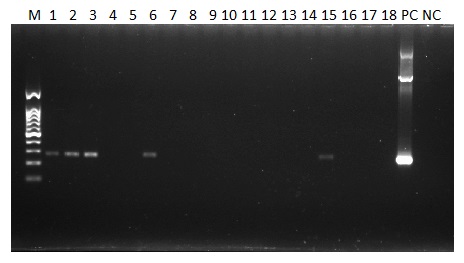


M: GenDirex 100 bp DNA ladder

Lane 1 – 18: Clinical samples of blood

PC: Positive control (*secY* plasmid)

NC: Non-template control

PCR amplified product: 276 bp size

300


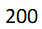


100

1. PCR of rat kidney samples


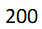

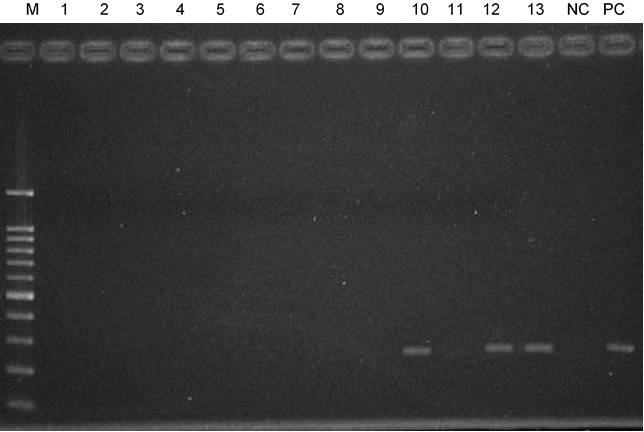


300

100

M: GenDirex 100 bp DNA ladder

Lane 1 – 13: Rat kidney samples

PC: Positive control (DNA from *Leptospira interrogans*)

NC: Non-template control

PCR amplified product: 276 bp size
