# Supplementary material for: A versatile isothermal amplification assay for the detection of leptospires from various sample types
Source: PeerJ. 2022 Mar 10;10:e12850. doi: 10.7717/peerj.12850 (PMC8918162; doi:10.7717/peerj.12850)
Supplement: Supplemental Information 8 [file peerj-10-12850-s008.docx]

EU357961.1 CCGGTTGTGGTTGCAGGAATCGCGAATGATCCTTCTTCCGAAGGTCTTCTCGGAATGGTG 60

EU358013.1 ----TTGTGGTTGCAGGAATCGCGAATGATCCTTCTTCCGAAGGACTTCTCGGAATGGTG 56

EU357956.1 CCGGTTGTGGTTGCAGGAATCGCGAATGATCCTTCTTCCGAAGGACTTCTCGGAATGGTG 60

EU357997.1 -----TGTGGTTGCAGGAATTGCGAATGATCCTTCTTCCGAAGGACTTCTCGGAATGGTG 55

EU358012.1 ----TTGTGGTTGCAGGAATCGCGAATGATCCTTCTTCCGAAGGTCTTCTCGGAATGGTA 56

*************** *********************** **************

EU357961.1 GATCTTTTTGCAGGTGGAGCTTTGTTGAAATTCTCCATCTTCGCGCTTGGAATCATGCCT 120

EU358013.1 GATCTTTTTGCAGGTGGAGCTTTGTTGAAATTCTCCATCTTCGCGCTTGGAATCATGCCT 116

EU357956.1 GATCTTTTTGCTGGCGGAGCTTTGTTGAAATTCTCCATCTTCGCGCTTGGAATCATGCCT 120

EU357997.1 GATCTTTTTGCTGGCGGAGCCTTGTTGAAATTTTCCATTTTCGCGCTTGGAATCATGCCT 115

EU358012.1 GATCTTTTTGCTGGCGGAGCCTTGTTGAAATTTTCCATCTTCGCGCTTGGAATCATGCCT 116

*********** ** ***** *********** ***** *********************

F3

EU357961.1 TATATTTCTTCTTCGATCGTAATGCAGCTTTTTATGGTCCTTGTTCCTGCCCTTCAAAAA 180

EU358013.1 TATATTTCTTCTTCGATCGTAATGCAGCTTTTTATGGTCCTTGTTCCTGCCCTTCAAAAA 176

EU357956.1 TATATTTCTTCTTCGATCGTAATGCAGCTTTTTATGGTCCTTGTTCCTGCCCTTCAAAAA 180

EU357997.1 TATATTTCTTCTTCGATCGTAATGCAGCTTTTTATGGTCCTTGTTCCTGCCCTTCAAAAA 175

EU358012.1 TATATTTCTTCTTCGATCGTAATGCAGCTTTTTATGGTCCTTGTTCCTGCCCTTCAAAAA 176

************************************************************

FIP

EU357961.1 CTTCAAAAAGAAGGAGAAGAAGGAAGAAAAAAAATTGGTCAATATACTAAGTATGGAACC 240

EU358013.1 CTTCAAAAAGAAGGAGAAGAAGGAAGAAAAAAAATTGGTCAATATACTAAGTATGGAACC 236

EU357956.1 CTTCAAAAAGAAGGAGAAGAAGGAAGAAAAAAAATCGGTCAATATACTAAGTATGGAACC 240

EU357997.1 CTTCAAAAAGAAGGAGAAGAAGGAAGAAAAAAAATCGGTCAATATACTAAGTATGGAACC 235

EU358012.1 CTTCAAAAAGAAGGAGAAGAAGGAAGAAAAAAAATCGGTCAATATACTAAGTATGGAACC 236

*********************************** ************************

FIP (cont.) LoopF FIP

EU357961.1 GTAATTCTTTGTGCGATTCAATCTTTAGCAGTGATCCAACTCGCAAAGGGCTGGTCTACC 300

EU358013.1 GTAATTCTTTGTGCGATTCAATCTTTAGCAGTGATCCAACTCGCAAAGGGCTGGTCTACC 296

EU357956.1 GTAATTCTTTGTGCGATTCAATCTTTAGCAGTGATCCAACTCGCAAAGGGCTGGTCTACC 300

EU357997.1 GTAATTCTTTGTGCGATTCAATCTTTAGCAGTGATCCAACTCGCAAAGGGCTGGTCTACC 295

EU358012.1 GTAATTCTTTGTGCGATTCAATCTTTAGCAGTGATCCAACTCGCAAAGGGCTGGTCTACC 296

************************************************************

FIP (cont.) BIP LoopB

EU357961.1 GGCACGGAACTTGAGCCTGCGCGTTACCCGGGCTTAATCAATTCTTCTGTTGTTCCTTAT 360

EU358013.1 GGCACGGAACTTGAGCCTGCGCGTTACCCGGGCTTAATCAATTCTTCTGTTGTTCCTTAT 356

EU357956.1 GGCACGGAACTTGAGCCTGCGCGTTACCCGGGCTTAATCAATTCTTCTGTTGTTCCTTAT 360

EU357997.1 GGCACGGAACTTGAGCCTGCGCGTTACCCGGGCTTAATCAATTCTTCTGTTGTTCCTTAT 355

EU358012.1 GGCACGGAACTTGAGCCTGCGCGTTACCCGGGCTTAATCAATTCTTCTGTTGTTCCTTAT 356

************************************************************

BIP B3

EU357961.1 TTTTATTTAATCGGAATCTTATCCATTACTACCGGAACCGTTCTTCTCATTTGGTTAGGA 420

EU358013.1 TTTTATTTAATCGGAATCTTATCCATTACTACCGGAACCGTTCTTCTCATTTGGTTAGGA 416

EU357956.1 TTTTATTTAATCGGAATCTTATCCATTACTACCGGAACCGTTCTTCTCATTTGGTTAGGA 420

EU357997.1 TTTTATTTAATCGGAATCTTATCCATTACTACCGGAACCGTTCTTCTCATTTGGTTAGGA 415

EU358012.1 TTTTATTTAATCGGAATCTTATCCATTACTACCGGAACCGTTCTTCTCATTTGGTTAGGA 416

************************************************************

B3 (cont.)

EU357961.1 GAACAGATCACCGAAAGAGGAATTGGTAACGGAATTTCTCTTTTGATCTTCGCTGGAATT 480

EU358013.1 GAACAGATCACCGAAAGAGGAATTGGTAACGGAATTTCTCTTTTGATCTTCGCTGGAATT 476

EU357956.1 GAACAGATCACCGAAAGAGGAATTGGTAACGGAATTTCTCTTTTGATCTTCGCTGGAATT 480

EU357997.1 GAACAGATCACCGAAAGAGGAATTGGTAATGGAATTTCTCTTTTGATCTTCGCTGGAATT 475

EU358012.1 GAACAGATCACCGAAAGAGGAATTGGTAACGGAATTTCTCTTTTGATCTTCGCTGGAATT 476

***************************** ******************************

EU357961.1 ATCGGAAGACTGCCTGAGTCTATGGTTCAACTTTTCTCTACCGATACGATGGATGCGTTG 540

EU358013.1 ATCGGAAGACTGCCTGAGTCTATGGTTCAACTTTTCTCTACCGATACGATGGATGCGTTG 536

EU357956.1 ATCGGAAGACTGCCTGAGTCTATGGTTCAACTTTTCTCTACCGATACGATGGATGCGTTG 540

EU357997.1 ATCGGAAGACTGCCTGAGTCTATGGTTCAACTTTTCTCTACCGATACGATGGATGCGTTG 535

EU358012.1 ATCGGAAGACTGCCTGAGTCTATGGTTCAACTTTTCTCTACCGATACGATGGATGCGTTG 536

************************************************************

EU357961.1 AATGTTCTTATCCTTTTGATCCTTTTTATTCTTCTCATTTCCTTGACGGTGTTATTAACG 600

EU358013.1 AATGTTCTTATCCTTTTGATTCTTTTTATTCTTCTCATTTCCTTGACGGTGTTATTAACG 596

EU357956.1 AACGTTCTTATCCTTTTGATTCTTTTTATTCTTCTCATTTCCTTGACGGTGTTATTAACG 600

EU357997.1 AATGTTCTTATCCTTTTGATTCTTTTTATTCTTCTCATTTCCTTGACGGTGTTATTAACG 595

EU358012.1 AATGTTCTTATCCTTTTGATTCTTTTTATTCTTCTCATTTCCTTGACGGTGTTATTAACG 596

** ***************** ***************************************

EU357961.1 CAAGGTGTGAGAAAGGTTCCTCTGCAGTACGGAAAACAAATGGTCGGAAGAAAAATGGTT 660

EU358013.1 CAAGGTGTGAGAAAGGTTCCTCTGCAGTACGGAAAACAAATGGTCGGAAGAAAAATGGTT 656

EU357956.1 CAAGGTGTGAGAAAGGTTCCTCTGCAGTACGGAAAACAAATGGTCGGAAGAAAAATGGTT 660

EU357997.1 CAAGGTGTGAGAAAGGTTCCTCTGCAGTACGGAAAACAAATGGTCGGAAGAAAAATGGTT 655

EU358012.1 CAAGGTGTGAGAAAGGTTCCTCTGCAGTACGGAAAACAAATGGTCGGAAGAAAAATGGTT 656
