# Supplementary material for: A versatile isothermal amplification assay for the detection of leptospires from various sample types
Source: PeerJ. 2022 Mar 10;10:e12850. doi: 10.7717/peerj.12850 (PMC8918162; doi:10.7717/peerj.12850)
Supplement: Supplemental Information 4 [file peerj-10-12850-s004.pdf]

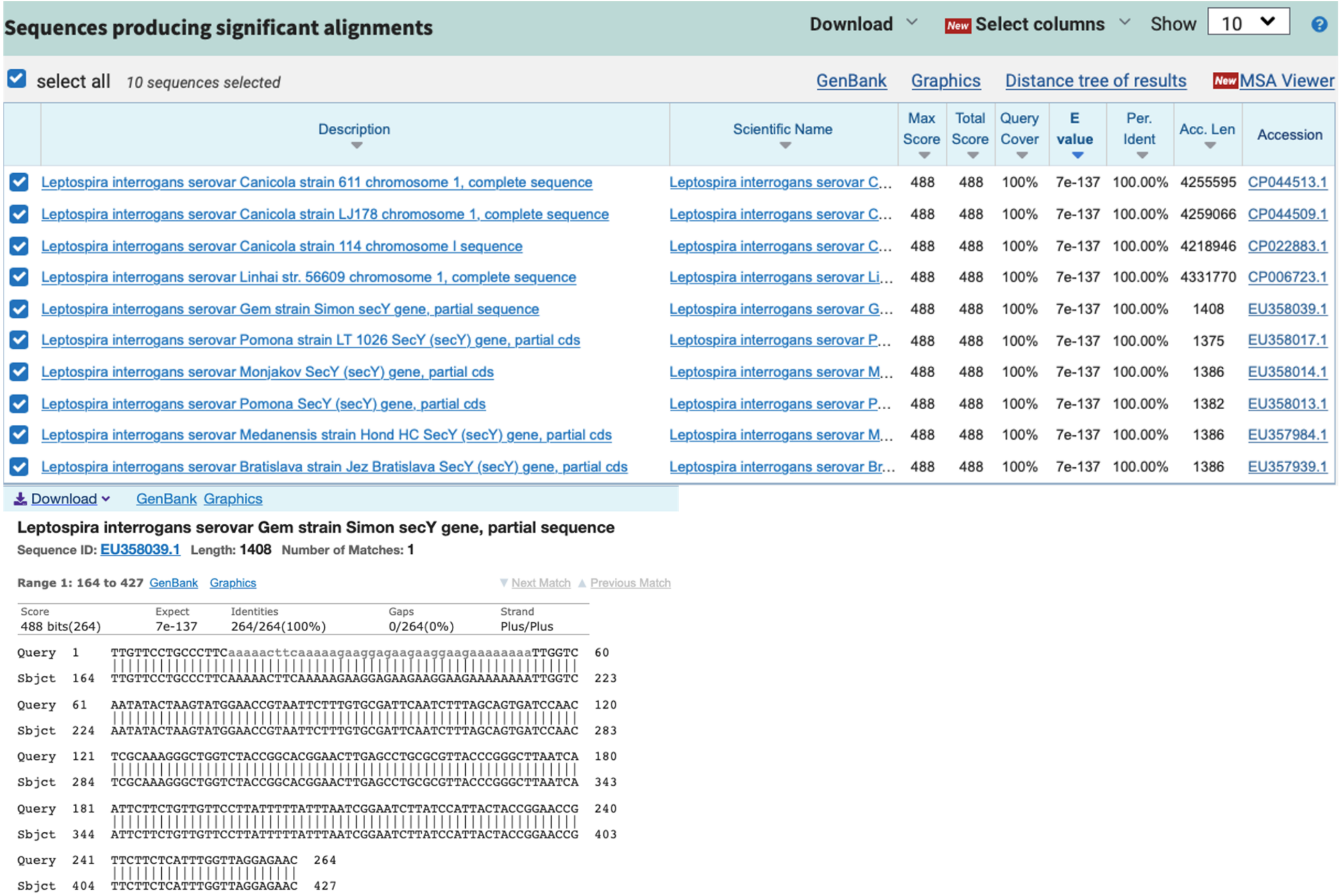

Figure S5. Sequencing and BLAST analysis of PCR reaction products from clinical urine samples (Sample 005).
